# Expression of p53, epidermal growth factor receptor, Ki-67 and O^6^-methylguanine-DNA methyltransferase in human gliomas

**DOI:** 10.3892/ol.2013.1317

**Published:** 2013-04-24

**Authors:** XINHUA HU, WEI MIAO, YUANJIE ZOU, WENBIN ZHANG, YANSONG ZHANG, HONGYI LIU

**Affiliations:** 1 Department of Neurosurgery, Brain Hospital Affiliated to Nanjing Medical University, Nanjing, Jiangsu 210029, P.R. China; 2 Department of Neurosurgery, Zhongda Hospital, Southeast University, Nanjing, Jiangsu 210029, P.R. China

**Keywords:** glioma, p53, Ki-67, epidermal growth factor receptor, O^6^-methylguanine-DNA methyltransferase

## Abstract

The present study aimed to evaluate the expression of p53, Ki-67, epidermal growth factor receptor (EGFR) and O^6^-methylguanine-DNA methyltransferase (MGMT), and to analyze the correlation between their expression and the histological grade of the tumors in 152 patients with gliomas. The tumors were classified according to the recommendations of the World Health Organization (WHO; 2007) into grade I (n=9), grade II (n=56), grade III (n=52) and grade IV (n=35). The expression of p53, Ki-67, EGFR and MGMT was analyzed using immunohistochemistry. The frequency of p53 immunopositivity was significantly lower in grade I gliomas than in grades II, III and IV. The frequency of EGFR immunopositivity was significantly higher in grade III and IV gliomas compared with grades I and II. The mean Ki-67 labelling index (LI) significantly increased in the higher glioma grades. The expression of MGMT in grade I and II tumors was not significantly different from that of grade III and IV tumors. The present data indicate that the expression of EGFR and Ki-67 is significantly correlated with the histological grade of the glioma, but that the expression of p53 and MGMT is not associated with the tumor grade.

## Introduction

Gliomas are the most common primary brain tumors in adults and have a poor survival outcome (1). Patients with the same tumor grades have highly variable prognoses, regardless of the therapeutic interventions that are used (2). Molecular behaviors have been observed to contribute to the varying prognoses of gliomas (3). p53, one of the most widely investigated molecules in human gliomas, has been shown to be a prognostic marker (4,5). Several studies have revealed that mutations in the epidermal growth factor receptor (EGFR) gene were associated with shorter intervals between relapses and a poorer survival rate for gliomas, and that EGFR expression is correlated with tumor progression (6,7). Ki-67, a marker of cell division, is a reliable indicator of tumor cell proliferative activity that has been associated with the histological grade and poor survival outcome for glioma (8). O^6^-methylguanine-DNA methyltransferase (MGMT) repairs damaged DNA and renders glioma cells resistant to alkylating agents. MGMT promoter methylation has been used as a biomarker to predict the sensitivity of gliomas to DNA alkylating chemotherapeutics (9).

Numerous studies have been undertaken to understand the molecular basis of gliomagenesis (10,11). However, to date, few studies have investigated the expression of p53, EGFR, Ki-67 and MGMT in the same group of glioma patients, particularly in the Chinese population. It has been reported that certain molecular markers are associated with the ethnicity of patients with glioma (10). Therefore, in order to understand the molecular features of gliomas with respect to ethnicity, the expression of these markers must be identified. In addition, the correlation between p53, EGFR, Ki-67 and MGMT expression and the histological grade of gliomas has not yet been established. The aim of the present study was to evaluate the expression of these markers using the immunohistochemical analysis of 152 tumor samples from Chinese patients with gliomas of various grades, and also to analyze the correlation between their expression and the tumor grades.

## Materials and methods

### Subjects

A total of 152 Chinese patients with gliomas (85 males and 67 females) were selected from the Nanjing Brain Hospital and Zhongda Hospital (Nanjing, Jiangsu, China) between June 2006 and June 2008. The mean age of the patients was 40.3 years (range, 21–79 years). Approval for the study was obtained from the institutional review board of Nanjing Brain Hospital and all patients provided informed consent.

The tumor grades were classified by two pathologists according to the recommendations of the World Health Organization (WHO; 2007) (14). The pathological grading of the cancers was as follows: grade I (5 cases of pilocytic astrocytoma, 3 of a subependymal tumor and 1 of subependymal giant cell astrocytoma), grade II (30 cases of astrocytoma, 8 of oligodendroglioma, 7 of ependymoma and 11 of small branch astrocytoma), grade III (33 cases of anaplastic astrocytoma and 19 of anaplastic oligoastrocytoma) and grade IV (24 cases of primary glioblastoma and 11 of secondary glioblastoma).

### Immunohistochemistry

Tissue sections (5-*μ*m thick) were obtained from formalin-fixed and paraffin-embedded tissue blocks for immunohistochemical staining. The samples were then incubated overnight in primary antibodies against p53, EGFR, Ki-67 and MGMT at 4°C. Mouse anti-human monoclonal primary antibodies against the following antigens were used: p53 (clone Do-7; 1:50 dilution); EGFR (clone EGFR.25; 1:50 dilution); Ki-67 (clone K-2; 1:100 dilution) and MGMT (clone MT3.1; 1:100 dilution). All primary antibodies were purchased from Ventana (Tucson, AZ, USA). Subsequent to the primary antibodies being washed off, the sections were incubated with goat anti-mouse biotin-conjugated secondary antibodies (1:1000 dilution; Ventana) for 20 min at 37°C. The tissue sections were then incubated with streptavidin horse-radish peroxidase for 20 min at 37°C. A 3,3′-diaminobenzidine (DAB) substrate was applied to the section for 10 min, prior to counter-staining with hematoxylin. The sections in which the primary antibodies were omitted were used as negative controls.

**Figure 1.**
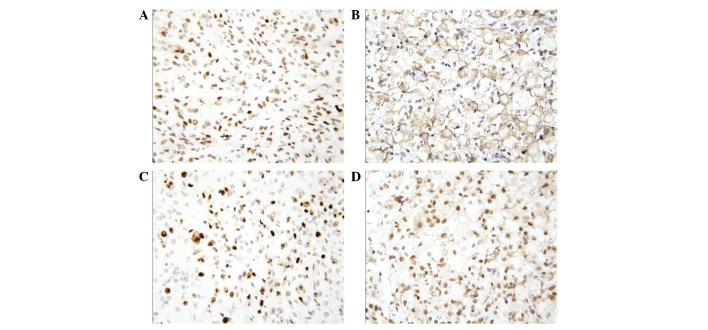
Immunostaining for p53, EGFR, Ki-67 and MGMT in human gliomas. (A) Representative p53 immunostaining showing nuclear staining in GB cells (magnification, ×400). (B) Representative EGFR immunostaining showing marked staining (>50% positive cells) in GB cells (magnification, ×400). (C) Representative Ki-67 immunostaining with Ki-67LI >40% in GB cells (magnification, ×400). (D) Representative MGMT immunostaining shows positive staining in GB cells (magnification, ×400). Scale bar: 50 *μ*m. EGFR, epidermal growth factor receptor; MGMT, O^6^-methylguanine-DNA transferase; GB glioblastoma.

The immunostaining was examined for the presence of p53, EGFR, Ki-67 and MGMT, and individually observed and counted by two independent neurosurgeons using a microscope. The presence of p53 and EGFR was determined using the percentage of immunostained cells per 200 cells in 5 fields. The p53 and EGFR scoring system (based on the number of positive cells) was as follows: negative (−), no positive cells observed in the random fields; weak positive (+), <25% positive cells; moderately positive (++), 25–50% positive cells; and strongly positive (+++), >50% positive cells. The labeling index (LI) for Ki-67 was calculated as the percentage of positive cells per 1,000 cells. The MGMT immunostained cells were classified as negative (<10%) or positive (≥10%; Fig. 1A–D).

### Statistical analysis

Statistical analysis was performed using SPSS 12.0 (SPSS Inc., Chicago, IL, USA). The χ^2^ test was performed to determine the significant differences observed in the p53, EGFR and MGMT expression values between the various glioma grades. An ANOVA was used to assess the significant differences observed in the Ki-67 LI between the glioma grades. P<0.05 was considered to indicate a statistically significant difference.

## Results

### Expression of p53 is lower in grade I gliomas

p53 was expressed in 2 out of 9 cases (22.2%) of grade I gliomas, 40 out of 56 cases (71.4%) of grade II gliomas, 38 out of 52 cases (73.1%) of grade III gliomas and 21 out of 35 cases (60.0%) of grade IV gliomas. The frequency of p53 immunopositivity was significantly lower in the grade I gliomas compared with the other three categories (P<0.05). No significant differences were identified in the frequency of p53 immunopositivity between the grade II, III and IV tumors (P=0.074; Table I).

### EGFR expression is associated with glioma grade

EGFR immmunopositive staining was observed in 2 cases (22.2%) of grade I glioma, 25 cases (44.6%) of grade II, 37 cases (71.2%) of grade III and 31 cases (88.6%) of grade IV. The frequency of EGFR immunopositivity was significantly higher in the grade III and IV gliomas than in the grade I and II gliomas (P=0.021, Table II). In addition, marked EGFR staining, ++ or +++, was observed in 40, 78.4 and 90.3% of grade II, III and IV immunopositive gliomas, respectively. EGFR expression was significantly higher in the higher grade gliomas compared with the lower grade gliomas (P=0.025).

### Ki-67 expression in the various glioma grades

**Figure 2.**
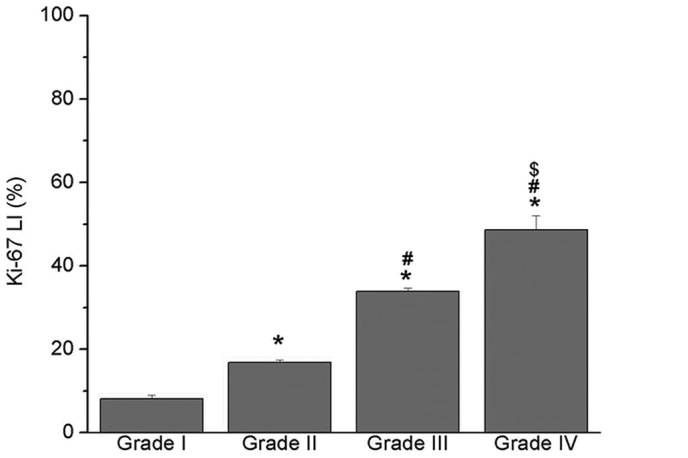
Labeling index (LI) for Ki-67 in grade I, II, III and IV gliomas. Ki-67 LI was measured as the percentage of positive cells per 1,000 cells. Results are presented as mean with SE bars. ^*^P<0.05 vs grade I, ^#^P<0.05 vs. grade II, ^$^P<0.05 vs. grade III.

The mean Ki-67 LI significantly increased with the glioma grade (Fig. 2). A significant difference was identified in the Ki-67 LI between the various glioma grades (P<0.05; Fig. 2), suggesting that the pathological grade was associated with the Ki-67 LI.

### MGMT expression is not associated with glioma grade

MGMT immunoreactivity was identified in 84 (55.3%) of the 152 glioma samples. The frequency of MGMT immunopositivity increased with the glioma grade (Table III). However, no significant differences were observed in the frequency of MGMT expression between the grades. In addition, the expression of MGMT in the grade I and II samples (30 out of 65 samples; 46.2%) was not significantly different from the expression in the grade III and IV tumors (54 out of 87 samples; 62.1%).

## Discussion

Despite developments in the diagnosis and treatment of gliomas, due to the quick progression of malignant tumors, the prognosis of affected patients remains poor. In order to accurately identify the factors that affect the prognosis of glioma patients and to select and evaluate the effectiveness of appropriate treatments, an understanding of the molecular mechanisms and the progression of gliomas is required. In the present study, immunohistochemistry was used to investigate the expression of p53, EGFR, Ki-67 and MGMT in 152 Chinese patients with gliomas, and to analyze their correlation with the histological glioma grade. The expression of EGFR and Ki-67 LI was observed to be significantly correlated with the histological grade of the gliomas, while the expression of p53 and MGMT was not associated.

p53 is one of the most frequently used molecular markers in gliomas. Immunocytochemical experiments have demonstrated that the overexpression of p53 is commonly considered to be a surrogate marker for p53 mutation (12). In the present study, it was observed that p53 expression was significantly higher in grade II gliomas than in grade I gliomas. Furthermore, p53 expression was marginally decreased in grade IV compared with grade II and III gliomas, suggesting that a p53 mutation may be an early event in glioma progression (13). The present data also revealed that no correlation existed between the expression of p53 and the tumor grade, suggesting that p53 expression is not a good prognostic marker for gliomas. However, the correlation between p53 immunoreactivity and the survival outcome of glioma patients remains controversial. It has been suggested that p53 may act as a weak independent prognostic marker for the clinical or pathological features of gliomas (5). This difference may be a result of the various methods used to detect p53 expression in glioma samples from differing patient populations. The ethnicity of patients with glioma appears to affect p53 expression in the various tumor grades (10). In addition, it has been noted that the changes in p53 overexpression in human astrocytic gliomas are generally associated with secondary, rather than primary glioblastomas (14). However, the present data are not consistent with this observation (data not shown). Furthermore, p53, a tumor suppressor gene, plays a key role in the cellular responses to various stresses. p53 also transfers cells with a normal p53 gene into tumor cells, sensitizing them to chemotherapeutic drugs and/or radiotherapy and promoting tumor cell apoptosis (15). In the present study, p53 expression was detected in approximately two-thirds of all 152 cases, suggesting that it may be an ideal target for glioma-targeted therapy.

The majority of gliomas express EGFR, which is often amplified, rearranged, mutated and/or overexpressed, particularly in malignant tumors (16). It has been reported that EGFR is closely correlated with tumor proliferation, metastasis, apoptosis, angiogenesis, sensitivity to radiotherapy and/ or chemotherapy and drug resistance (17). A previous study has shown that glioma malignancy increases with EGFR amplification and overexpression (18). Consistent with this, the present study showed that EGFR overexpression was evident in 25 cases (44.6%) of grade II, 37 cases (71.2%) of grade III and 31 cases (88.6%) of grade IV tumors. However, the frequency of EGFR expression shown in the present data was inconsistent with another study stating that EGFR amplification was identified in 40–50% of glioblastomas and ∼10% of anaplastic astrocytomas, but not in low-grade astrocytomas (19). This difference may have been a result of the dissimilar patient populations in the two studies. However, the two studies did each reveal that EGFR overexpression is significantly greater in high-grade gliomas than in low-grade tumors. In addition, EGFR activation has been shown to be a significant indicator of glioma deterioration, as it is a vital marker of poorly-differentiated gliomas (17–20). In the present study, EGFR overexpression correlated with the higher grade gliomas, suggesting that EGFR overexpression was associated with tumor aggressiveness and invasion. However, the role of EGFR overexpression in the prognosis of gliomas remains controversial. It has been observed that EGFR amplification does not significantly affect the survival of patients with glioblastoma at any age (21). However, a region-limited and age-limited study has observed that EGFR amplification and overexpression is a significant predictor of survival (7).

The levels of Ki-67, a cell proliferation nuclear antigen, may objectively reflect the proliferation and malignancy of tumor cells (22). Increased Ki-67 expression has been shown to positively correlate with the increased grade of malignancy and a poor prognosis in glioma patients (23). In agreement with these studies, the present data showed that a substantial increase in Ki-67 expression was correlated with a higher tumor grade, suggesting that Ki-67 expression is a good marker for glioma malignancy. In addition, Ki-67 is a significant marker for differentiating between benign and malignant tumors. When Ki-67 is overexpressed, proliferation and invasiveness increase, resulting in tumor recurrence and malignant changes (24).

MGMT is involved in the repair of DNA damage and the prevention of second-level DNA damage, thus rendering glioma cells resistant to DNA alkylating agents. The detection of MGMT methylation does not provide useful information with regard to prognosis, but may predict whether patients with glioblastomas are able to benefit from temozolomide therapy (25). Methylation of the MGMT promoter may be detected in 60% of glioblastomas, although the false-positive rate is high (25). The expression levels of MGMT mRNA and protein are significantly correlated with enzyme activity. The survival time for patients who are negative for MGMT is longer than that of patients who are positive for MGMT (26). In the present study, it was revealed that MGMT was expressed in 55.3% of glioma samples. The expression of MGMT was increased in the high-grade gliomas (62.1%) compared with the low-grade gliomas (46.2%), suggesting that the majority of gliomas (particularly malignant gliomas) are not sensitive to chemotherapy. The majority of studies have focused on MGMT expression in high-grade gliomas, but few have explored MGMT expression in the other grades. Yang *et al* (27) showed that MGMT expression correlates with the glioma grade. However, the present data identified no significant differences between MGMT expression and the various glioma grades. This difference may have been due to the dissimilar sample sizes and detection methods that were employed.

In summary, the expression of p53, EGFR, Ki-67 and MGMT was investigated in gliomas in a Chinese population using immunocytochemistry. It was identified that the expression of EGFR and Ki-67 LI, but not p53 and MGMT, correlated with the histological grade of the gliomas. The present study contributes to the assessment of the invasiveness and proliferative potential of gliomas, to predict their sensitivity to chemotherapy and/or radiotherapy and to determine the effectiveness of molecular-targeted therapy. Further studies are required to understand the molecular biological behavior of malignant gliomas.

## Figures and Tables

**Table I.** p53 immunoreactivity in human gliomas.

Grade	Immunonegative cases, n (%)	Immunopositive cases, n (%)			
		+	++	+++	Total
I	7 (77.8)	1 (11.1)	1 (11.1)	0 (0.00)	2 (22.2)
II	16 (28.6)	11 (19.6)	12 (21.4)	17 (30.4)	40 (71.4)
III	14 (26.9)	11 (21.2)	11 (21.2)	16 (30.8)	38 (73.1)
IV	14 (40.0)	3 (8.6)	6 (17.1)	12 (34.3)	21 (60.0)

P<0.05, grade I vs. II, III, and IV (χ^2^ test).

**Table II.** EGFR immunoreactivity in human gliomas

Grade	Immunonegative cases, n (%)	Immunopositive cases, n (%)			
		+	++	+++	Total
I	7 (77.8)	2 (22.2)	0 (0.0)	0 (0.0)	2 (22.2)
II	31 (55.4)	15 (26.8)	6 (10.7)	4 (7.1)	25 (44.6)
III	15 (28.8)	8 (15.4)	17 (32.7)	12 (23.1)	37 (71.2)
IV	4 (11.4)	3 (8.6)	10 (28.6)	18 (51.4)	31 (88.6)

P=0.021, grade I and II vs. grade III and IV (χ^2^ test). EGFR, epidermal growth factor receptor.

**Table III.** MGMT immunoreactivity in human gliomas.

Grade	Immunonegative cases, n (%)	Immunopositive cases, n (%)
I	6 (66.7)	3 (33.3)
II	29 (51.8)	27 (48.2)
III	22 (42.3)	30 (57.7)
IV	11 (31.4)	24 (68.6)

P>0.05 among grades I, II, III and IV (χ^2^ test). MGMT, O^6^-methylguanine-DNA methyltransferase.

## References

[1] Kogiku M, Ohsawa I, Matsumoto K (2008). Prognosis of glioma patients by combined immunostaining for survivin, Ki-67 and epidermal growth factor receptor. J Clin Neurosci.

[2] van den Bent MJ, Kros JM (2007). Predictive and prognostic markers in neuro-oncology. J Neuropathol Exp Neurol.

[3] Noble M, Dietrich J (2004). The complex identity of brain tumors: emerging concerns regarding origin, diversity and plasticity. Trends Neurosci.

[4] Kyritsis AP, Bondy ML, Hess KR (1995). Prognostic significance of p53 immunoreactivity in patients with glioma. Clin Cancer Res.

[5] Levidou G, El-Habr E, Saetta AA (2010). P53 immunoexpression as a prognostic marker for human astrocytomas: a meta-analysis and review of the literature. J Neurooncol.

[6] Hoelzinger DB, Mariani L, Weis J (2005). Gene expression profile of glioblastoma multiforme invasive phenotype points to new therapeutic targets. Neoplasia.

[7] Shinojima N, Tada K, Shiraishi S (2003). Prognostic value of epidermal growth factor receptor in patients with glioblastoma multiforme. Cancer Res.

[8] Montine TJ, Vandersteenhoven JJ, Aguzzi A (1994). Prognostic significance of Ki-67 proliferation index in supratentorial fibrillary astrocytic neoplasms. Neurosurgery.

[9] von Deimling A, Korshunov A, Hartmann C (2011). The next generation of glioma biomarkers: MGMT methylation, BRAF fusions and IDH1 mutations. Brain Pathol.

[10] Wiencke JK, Aldape K, McMillan A (2005). Molecular features of adult glioma associated with patient race/ethnicity, age, and a polymorphism in O6-methylguanine-DNA-methyltransferase. Cancer Epidemiol Biomarkers Prev.

[11] Fan KJ, Pezeshkpour GH (1992). Ethnic distribution of primary central nervous system tumors in Washington, DC, 1971 to 1985. J Natl Med Assoc.

[12] Srivastava P, Jaiswal PK, Singh V, Mittal RD (2011). Role of p53 gene polymorphism and bladder cancer predisposition in northern India. Cancer Biomark.

[13] Pardo FS, Hsu DW, Zeheb R, Efird JT, Okunieff PG, Malkin DM (2004). Mutant, wild type, or overall p53 expression: freedom from clinical progression in tumours of astrocytic lineage. Br J Cancer.

[14] Louis DN, Ohgaki H, Wiestler OD (2007). The 2007 WHO classification of tumours of the central nervous system. Acta Neuropathol.

[15] Bourdon JC, Laurenzi VD, Melino G, Lane D (2003). p53: 25 years of research and more questions to answer. Cell Death Differ.

[16] Nicholas MK, Lukas RV, Jafri NF, Faoro L, Salgia R (2006). Epidermal growth factor receptor - mediated signal transduction in the development and therapy of gliomas. Clin Cancer Res.

[17] De Luca A, Carotenuto A, Rachiglio A (2008). The role of the EGFR signaling in tumor microenvironment. J Cell Physiol.

[18] Wang A, Li J, Huang Q (2009). Preliminary studies on the target inhibition effect of epidermal growth factor receptor inhibitor on proliferation of glioma cells. J Int Neurol Neurosurg.

[19] Hofer S, Lassman AB (2010). Molecular markers in gliomas: impact for the clinician. Target Oncol.

[20] Ambroise MM, Khosla C, Ghosh M, Mallikarjuna VS, Annapurneswari S (2010). The role of immunohistochemistry in predicting behavior of astrocytic tumors. Asian Pac J Cancer Prev.

[21] Ohgaki H, Dessen P, Jourde B (2004). Genetic pathways to glioblastoma: a population-based study. Cancer Res.

[22] Gerdes J, Li L, Schlueter C (1991). Immunobiochemical and molecular biologic characterization of the cell proliferation-associated nuclear antigen that is defined by monoclonal antibody Ki-67. Am J Pathol.

[23] Johannessen AL, Torp SH (2006). The clinical value of Ki-67/MIB-1 labeling index in human astrocytomas. Pathol Oncol Res.

[24] Habberstad AH, Gulati S, Torp SH (2011). Evaluation of the proliferation markers Ki-67/MIB-1, mitosin, survivin, pHH3, and DNA topoisomerase IIα in human anaplastic astrocytomas - an immunohistochemical study. Diagn Pathol.

[25] Stupp R, Hegi ME, Mason WP (2009). Effects of radiotherapy with concomitant and adjuvant temozolomide versus radiotherapy alone on survival in glioblastoma in a randomised phase III study: 5-year analysis of the EORTC-NCIC trial. Lancet Oncol.

[26] Nakasu S, Fukami T, Jito J, Matsuda M (2007). Prognostic significance of loss of O6-methylguanine-DNA methyltransferase expression in supratentorial diffuse low-grade astrocytoma. Surg Neurol.

[27] Yang Z, Deng Y, Fang J (2008). Expressions of LRP, MGMT and Topo IIα in brain glioma and normal brain tissue and their significances. J Clin Res.

